# Miodynamic and Radiographic Evaluation in Recreative Athletes with Patellofemoral Pain

**DOI:** 10.3390/medicina60111860

**Published:** 2024-11-13

**Authors:** Abiel Eugenio Garza-Borjón, Mirna González-González, José Fernando de la Garza-Salazar, Mario Simental-Mendía, Carlos Acosta-Olivo

**Affiliations:** 1 Escuela de Medicina y Ciencias de la Salud, Tecnológico de Monterrey, Monterrey 64700, Mexico; dr.abielgb@garzaborjon.com (A.E.G.-B.); mirnagonzalez@tec.mx (M.G.-G.); 2 Institute of Orthopedics and Traumatology, Tecnológico de Monterrey, San Pedro Garza García 66278, Mexico;; 3 Service of Orthopedics and Traumatology, Hospital Universitario “Dr. José E. González”, Universidad Autónoma de Nuevo León, Monterrey 64460, Mexico; mario.simentalme@uanl.edu.mx

**Keywords:** patellofemoral pain, muscle strength, aerobic activity, anterior knee pain

## Abstract

*Background and Objectives:* Patellofemoral pain (PFP) is frequent in the young and active population. The effect of muscle strength in the lower extremities after aerobic activity in patients with this condition has yet to be detailed. Our objective was to determine if patients with PFP show alterations in lower extremity muscle strength measurements after performing a session of ten minutes of aerobic activity on a treadmill compared to people without patellofemoral pain. *Materials and Methods:* We conducted a prospective experimental study with a stratified, non-randomized, and non-blinded population sample with group matching, including an experimental group with PFP and a control group with no pain. Subjects completed self-reported functional questionnaires (IKDC, Kujala, KOOS, SF-12), underwent radiographic studies, and were evaluated by measuring the strength of hip and knee muscles and the Single-Leg Triple-Hop (SLTH) test before and after ten minutes of exercise on a treadmill. *Results:* Seventeen subjects diagnosed with PFP and seventeen control subjects were evaluated. Both groups were homogeneous and had no significant differences in the demographic variables. A wider sulcus angle at 30° (136.8 ± 3.8° vs. 132.5 ± 5.6°, *p* = 0.0140), a decrease strength post-exercise in the hip abductor (37.9 ± 7.1 N⋅m vs. 45.6 ± 7.7 N⋅m, *p* < 0.05) and knee extensor (36.0 ± 9.1 N⋅m vs. 47.7 ± 14.0 N⋅m, *p* < 0.05), and a shorter distance in the SLTH test (337.9 ± 74.9 cm vs. 438.6 ± 65.8 cm, *p* < 0.01) was recorded in subjects with patellofemoral pain. *Conclusions:* Subjects with PFP had an overall lower strength of hip and knee muscles, showing significant differences in the hip abductors and knee extensors between people with PFP and healthy matched controls after aerobic exercise.

## 1. Introduction

Patellofemoral pain (PFP) was first described in 1928 [1]. It is characterized by an insidious pain around the patella, which is often associated with overuse or misuse of the limb and has a high prevalence of up to 45% of the population [2]. PFP symptoms typically worsen during load-bearing activities or when maintaining prolonged knee flexion [3,4,5,6]. Unlike chondromalacia, which involves chondral softening detectable by imaging studies or arthroscopy, PFP lacks these specific tissue changes [7]. Imaging studies, such as X-Rays, computed tomography scans, and magnetic resonance imaging, can be employed to exclude other diagnoses, assess disease progression, and identify anatomical or mechanical factors that may predispose the lower extremity to functional alterations [8]. Although PFP is generally considered benign and self-limiting, only 33% of patients are asymptomatic after two years [2,3,9]. The pain associated with PFP has been linked to alterations in the anatomical and mechanical structures involved in knee mobility and stability. The increasing popularity of sports, particularly running, has intensified interest in the prevention of this condition [10]. Among novice runners, 17–21% develop PFP early in their training, often forcing them to stop within a 29-min run [3,11]. This phenomenon has been associated with alterations in lower-extremity myodynamics, which can lead to patellar malposition, compression of the patellar articular facet, impaired proprioception, and soft tissue inflammation [6,9,12,13,14,15,16].

Previous research has shown that strengthening weak muscle groups can relieve PFP symptoms, yet the immediate muscular strength response to physical activity in PFP patients remains unexplored [4,6,17]. The objective of this study was to assess baseline muscle strength and evaluate its response after aerobic physical activity to determine whether there is a tendency toward muscular balance or a more pronounced alteration in strength ratios. We hypothesized that individuals with PFP would exhibit altered myodynamic parameters in hip abductors, knee flexors, knee extensors, and the Hamstring/Quadriceps (H/Q) ratio, with these alterations becoming more pronounced following physical activity compared to individuals without PFP.

## 2. Materials and Methods

### 2.1. Study Design

This study was a clinical trial with a bivariable correlation and a stratified, non-randomized, non-blinded sample with group matching. It was conducted from September to November 2023, following approval by the TecSalud, Hospital Zambrano Hellion’s Research Ethics Committee and Research Committee.

### 2.2. Eligibility Criteria

The inclusion criteria for the experimental group were male and female patients aged 18 to 40 years, with a body mass index (BMI) between 18.5 and 29.9, experiencing anterior knee pain for at least 14 days without previous medical management, and a clinical examination confirming a diagnosis of PFP, along with informed consent. The control group included individuals with similar characteristics (age and BMI) but without anterior knee pain, confirmed through clinical examination.

Participants were excluded if they had congenital or acquired angular deformities, lower extremity surgeries, metabolic disease, pregnancy, physical activity restrictions due to comorbid conditions, or abnormal vital signs before testing. Patients unable to complete the intervention or physical test due to general discomfort or scoring above 10% on the Globo Risk Score for cardiovascular risk were also excluded [18]. As in the study by Ireland et al., [16]. the affected knee was evaluated, and for those with bilateral symptoms, the extremity with more significant pain was considered. Patients with a BMI indicating underweight or overweight were excluded to avoid confounding effects, as being overweight can predispose individuals to knee pain and osteoarthritis, while being underweight can indicate nutritional disorders associated with fracture risk, cardiovascular diseases, and increased mortality [19,20].

### 2.3. Procedure

Four assessments were conducted during a single clinic visit, covering anthropometric variables, patient-reported outcomes, radiographic imaging for articular and mechanical angles, and a strength test for various muscle groups to observe changes following aerobic exercise. Each evaluation is detailed below.

### 2.4. Anthropometric Evaluation

Physical characteristics were recorded using the following methods. BMI was calculated based on each participant’s weight and height. Additionally, thigh circumference was measured at three locations: at the superior pole of the patella and 5 and 10 cm above this point. These measurements were used to verify group comparability for subsequent evaluations.

### 2.5. Patient-Reported Outcomes

The Knee Injury and Osteoarthritis Outcome Score (KOOS) assesses five domains: pain, symptoms, function in daily life, function in sports/recreation, and quality of life. It includes forty-two questions, scored from zero (no problem) to four (severe limitation). The final score ranges from 0 to 100, with zero indicating severe knee issues and 100 representing optimal knee health [21].

The International Knee Documentation Committee (IKDC) questionnaire evaluates symptoms, sports activity, and knee function. Scores range from 0 to 100, where 100 represents optimal knee function [22]. The Kujala scale assesses thirteen aspects of PFP, with answers scored from 0 to 10. A higher final score (0 to 100) indicates fewer symptoms and greater functionality [23]. The Short Form Health Survey (SF-12) measures both physical and mental health across eight areas: physical function, physical limitations, bodily pain, general health, vitality, social functioning, emotional limitations, and mental health. Results are divided into mental and physical component scores, with a score of 50 (±10) indicating a range that is considered normal [24].

### 2.6. Radiographic Evaluation

Imaging studies were performed using the Optima XR 646 (Ronkonkoma, NY, USA. Model 2013, General Electric) DRX Excel Plus and analyzed with the Carestream Stitching Module software Version 12.2.2.1025. All radiographs were taken by the same technician, and a single trained radiologist measured the angles three times to obtain an average.

**Figure 1:**
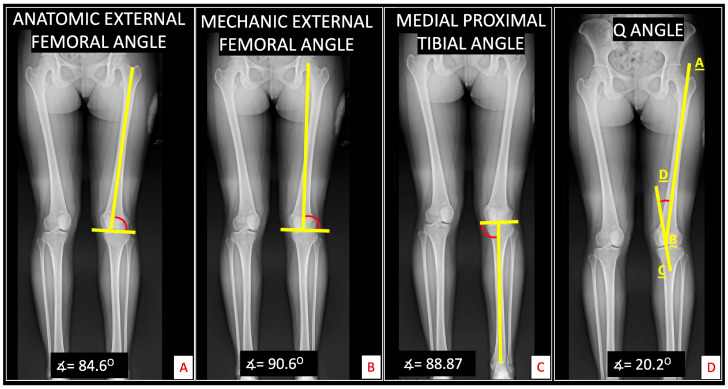
Radiographic evaluation in the panoramic projection. Panoramic lower extremity X-Ray was used to measure (**A**) the anatomic external femoral angle, with tolerable values between 79° and 93°; (**B**) the mechanic external femoral angle, with tolerable values between 85° and 90°; (**C**) the medial proximal tibial angle, with tolerable values between 85° and 90°; and (**D**) Q angle, with tolerable values between 8–15° for women and 8–10° for men. Obtained by the angle formed by the intersection of lines AB (Anterior superior iliac spine- Center of patella) and CD (Tibial Tuberosity-Line passing the center of patella).

First, a panoramic projection of the lower extremities was used to measure the anatomical external femoral angle. This angle is determined by drawing a line through the center of the femoral diaphysis to its intersection with a line passing through the lowest point of both femoral condyles, with the superior and external angle recorded. The mechanical external femoral angle was measured on the same radiograph by drawing a line from the center of the femoral head to the femoral condyles’ center, intersecting a line through the lowest points of both condyles [25]. The medial proximal tibial angle (MPTA) was obtained by drawing a line along the tibia’s mechanical axis and intersecting it with a line through the tibial plateau’s superior medial portion [26]. Finally, the Q angle was measured by drawing a line from the anterior superior iliac spine to the patella’s center and intersecting it with a line from the tibial tuberosity to the patella’s center [27] (Figure 1).

**Figure 2:**
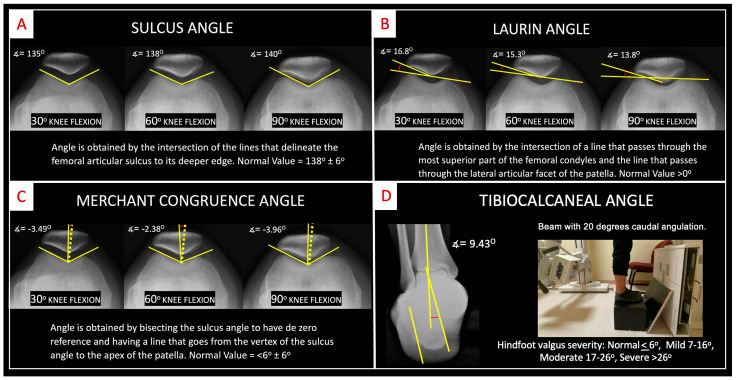
Radiographic evaluation of knee axial projections. Knee axial X-Rays with 30°, 60°, and 90° of flexion and hindfoot X-Ray of the ankle to obtain (**A**) sulcus angle; (**B**) Laurin angle; (**C**) Merchant congruence angle; and (**D**) the hindfoot alignment X-Ray view with measurement of the tibiocalcaneal angle.

Axial knee projections at 30°, 60°, and 90° were taken to assess the sulcus angle, Laurin’s lateral patellofemoral angle, and Merchant’s congruence angle [28]. A hindfoot alignment X-Ray was also performed to evaluate the coronal alignment of the hindfoot through the tibio–calcaneal angle. This angle is determined by drawing a line along the tibial diaphysis and two additional lines through the medial and lateral margins of the calcaneus [29,30] (Figure 2).

### 2.7. Muscle Strength Evaluation and Single-Leg Triple-Hop (SLTH) Test

The maximum muscle strength of the hip flexors, abductors, and adductors and knee flexors and extensors was evaluated. Measurements were recorded in Newton-meters (N⋅m) using the MicroFET 2 Wireless hand-held dynamometer (Hoggan Health Industries; West Jordan, UT, USA) [31,32]. The hand-held dynamometer is a reliable tool, showing minimal differences compared to the isokinetic dynamometer [33]. Values were normalized by body weight following the formula from Robinson et al. [6]. Each subject’s affected leg was tested three times, with each trial lasting three seconds. For participants with bilateral symptoms, the leg with more significant limitations was selected. In the control group, the right leg was used for comparative evaluation. The measurement techniques were based on those used in studies assessing the same muscle groups. [9,16,32].

**Figure 3:**
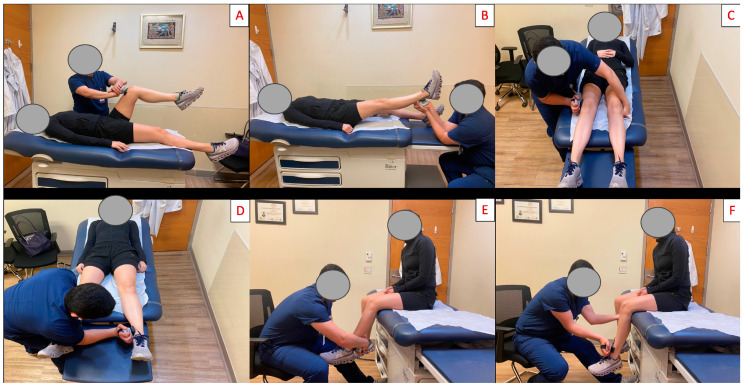
Muscle strength evaluation. Measurements were obtained with a MicroFET 2 wireless hand-held dynamometer. (**A**) Hip flexor test; (**B**) hip extensor test; (**C**) hip abduction test; (**D**) hip adduction test; (**E**) knee flexor test; and (**F**) knee extension test.

To measure hip flexor strength, the patient laid supine with the hip and knee flexed at 90°, resisting while flexing the hip against the dynamometer. For knee extensor strength, the examiner positioned the elbow against a rigid surface as support; the patient placed their ankle in the dynamometer with the leg fully extended and was instructed to extend the entire leg. Hip abductor strength was measured with the patient in a supine position, hip and knee in neutral, and the dynamometer positioned above the lateral epicondyle while the patient attempted to abduct the leg. Hip adductor strength was measured by placing the forearm between the medial malleoli with the hip and knee in neutral; the patient was instructed to adduct against the dynamometer. For knee flexion strength, the patient sat on the edge of the examination table with the knee and hip flexed at 90°, and the dynamometer was placed above the calcaneus as the patient contracted the flexor muscles. Finally, knee extensor strength was assessed with the patient seated, hip and knee flexed at 90°, and the dynamometer placed on the anterior tibia above the malleolar level; the patient activated the quadriceps to extend the knee (Figure 3).

After the initial muscle measurements, participants performed the Single-Leg Triple-Hop (SLTH) test, which measured the total distance covered in three consecutive hops using only the designated leg. Following this, participants completed a 10-min aerobic exercise. The duration was selected based on evidence that aerobic activities lasting more than 7 min yield significant health benefits [34]. Participants jogged for 10 min on a Life Fitness 95T treadmill. The first minute, at 3 km per hour (km/h), served as a warm-up. For the subsequent minutes, the speed increased to 8 km/h, and the incline level was raised by one unit at minutes one, four, and seven. The final minute was designated for cool-down, with the incline decreasing by one unit every 20 s and the speed reduced to 5 km/h.

**Figure 4:**
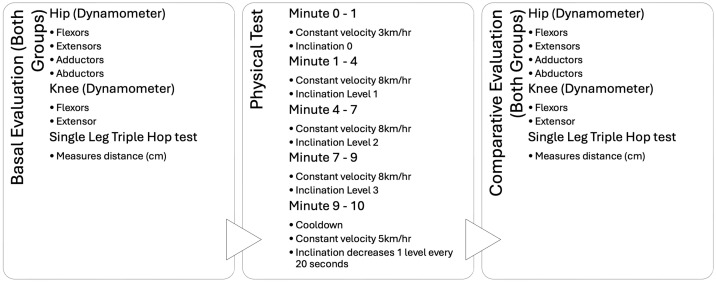
Aerobic test diagram. Summary of the aerobic physical test procedure and the variables measured.

During the test, participants were asked to report any onset of symptoms to ensure the exercise intensity was sufficient to provoke them if present. After the treadmill run, muscle strength measurements were repeated using the same technique as before, along with the post-SLTH test. Figure 4 outlines the aerobic test procedure and the evaluations conducted for each participant.

### 2.8. Sample-Size Calculation

Based on prior results from a study evaluating isometric hip strength in female subjects with patellofemoral joint pain compared to a control group [16], we estimated a representative sample size using a formula for the difference between two means, with a power of 95%, a confidence level of 95%, and a two-sided α-value of 1.96. This calculation indicated that 15 participants per group were needed to detect a significant difference in the myodynamic characteristics measured.

### 2.9. Statistical Analysis

Unless otherwise stated, all data are reported as mean ± standard deviation with 95% confidence intervals. The Kolmogorov–Smirnov test was used to assess the normal distribution of all variables included in this study. For quantitative variables with normal distribution, such as demographic, anthropometric, and radiological measures, an unpaired *t*-test was used to compare study groups. The Mann–Whitney U test was applied for non-normally distributed variables (e.g., clinical scores). To compare quantitative data within and between groups across all myodynamic evaluations, a one-way analysis of variance (ANOVA) with Tukey’s multiple comparison test was performed. A *p*-value < 0.05 was considered statistically significant. Statistical analyses were conducted using GraphPad Prism 5.00 software for Windows (GraphPad Software, San Diego, CA, USA).

## 3. Results

### 3.1. Patient Selection and Demographic Characteristics

Seventeen patients met the inclusion criteria and completed the study. An equal number of healthy subjects were recruited to form the control group, ensuring a matched sample. All participants in both groups signed informed consent forms and completed the physical tests without complications. Both groups had an equal distribution of female and male patients and were homogeneous in terms of demographic characteristics (Table 1).

### 3.2. Anthropometric Analysis

The anthropometric variables measured in both groups showed no significant differences, except for thigh circumference at the superior pole of the patella, where the PFP group had a larger measurement. Table 2 presents the anthropometric variables for both groups.

### 3.3. Patient-Reported Outcomes

All questionnaires assessing clinical symptoms and limitations during recreational, sports, daily living activities, and quality of life indicated significantly better scores in the control group. The mental health evaluation from the SF-12 showed no significant differences between the two groups (Table 3).

### 3.4. Radiographic Evaluation

All the radiographic angles measured had no significant differences between groups except for the sulcus angle measured at 30°, which had a wider angle in the PFP group (Table 4).

### 3.5. Muscle Strength and SLTH Test Evaluation

Post-test measurements indicated that the strength of the hip abductors and knee extensors was significantly greater in the control group, and the Single-Leg Triple-Hop (SLTH) test scores were also notably higher. The remaining variables exhibited non-significant differences. While both groups showed a general tendency towards increased muscle strength in the post- vs. pre-analysis tests, the hip abductors in the PFP group experienced a reduction in strength.

Pre-test values between the control and PFP groups were analyzed, revealing no significant differences; however, all values were higher in the control group except for the Hamstrings/Quadriceps (H/Q) ratio, which was greater in the PFP group. The post-test comparison demonstrated significant differences in hip abductor strength, knee extensor strength, and SLTH test performance, with the control group exhibiting greater strength and distance traveled. Additionally, the aerobic test induced pain in all patients in the PFP group, with an average duration of 413.2 ± 198.2 s. Table 5 presents the comprehensive muscle evaluations for both groups.

## 4. Discussion

Patellofemoral pain (PFP) causes discomfort in daily living and sports-related activities, leading to diminished scores on clinical scales. It has been associated with malalignment, muscle imbalances, and overload, all of which can influence the onset and persistence of symptoms [7,32,35]. Malalignment refers to the relationship between the patella and the trochlear groove, which can be classified as central, lateral, medial, high, or low. This positioning determines whether the facets of the patella experience areas of stress or overload. Muscle imbalances may affect the biomechanics and alignment of the joint; for instance, a weaker abductor could predispose to lateral displacement of the patella and an increased Q angle. Overload pertains to the patient’s activities that may contribute to stress on the joint. Evaluating these three areas related to PFP may help identify the factors contributing to this pathology [35,36]. Patients with PFP exhibited notable weakness in hip and knee muscles after 10 min of aerobic exercise.

Physical activity, particularly focused on strength training, is an integral part of managing PFP. Various authors report that just seven minutes of daily exercise can result in significant metabolic and physical benefits [34]. Gallagher et al. [37] identified appropriate exercise intensities with walking speeds of 4 km/h for flat terrain, 5.6 km/h for walking on an incline, and 8 km/h for running. The data analyzed post-aerobic exercise reflected an increase in the alterations initially observed, which may predispose individuals to mechanical imbalances and more intense symptoms. This information could be instrumental in developing a management plan for PFP [9,16,32,38,39,40].

Interestingly, only one measurement of thigh circumference was significantly greater in patients with PFP, specifically above the patella. This may indicate inflammation of the suprapatellar bursa or intraarticular synovitis, which could alter joint function and serve as a valuable risk factor in the PFP population [41,42]. Ultrasound could be an excellent tool to support this hypothesis, as it avoids radiation, is non-invasive, and could be readily available in clinical settings to evaluate muscle thickness, tendinosis, the presence of bursitis, and effusion [43].

On average, significant differences were found in the IKDC, Kujala, and KOOS scores, with the quality of life category being the most affected domain. These differences highlight how drastically patient functionality is compromised compared to the general population, indicating that individuals with this condition experience setbacks relative to their peers. The SF-12 questionnaire significantly revealed physical limitations, although the mental health evaluation, while lower than that of the control group, did not show significant differences. Many patients report feelings of frustration, low mood, and low self-esteem, as noted by several authors [12,39]. However, the questionnaire results do not provide sufficient justification to prioritize psychological management as a primary intervention. All patients in the PFP group engaged in recreational sports activities; none required sports participation as part of their daily responsibilities. This distinction emphasizes that these activities serve more as hobbies for health maintenance rather than essential tasks, which may mitigate potential impacts on mental stability. Nonetheless, it is advisable to incorporate mental assessments to rule out associated pathologies.

The Merchant angle measurements remained within acceptable ranges, showing no significant alterations. However, the sulcus angle at 30° was significantly greater in the PFP group, while the other angles did not exhibit substantial differences. A wider sulcus angle at 30° is associated with trochlear dysplasia, which can increase the risk of patellofemoral instability and PFP by up to 11 times [44]. At this angle, the patella experiences higher chondral pressure on the femoral groove, potentially leading to overload and triggering symptoms [39]. The Laurin angle, indicative of patellar tilt, remained within normal ranges for both groups. The tibial–calcaneal angle in both groups fell into the “mild” category of rearfoot valgus, with no significant differences noted. However, the PFP group exhibited an average of 2.3° more than the control group, approaching the classification of “moderate” rearfoot valgus [45]. Despite this, there was no correlation between rearfoot alignment and patellofemoral pain, as both groups were homogeneous.

Results from the radiographic evaluation, including the mechanical external femoral angle, anatomical femoral angle, medial proximal tibial angle, and Q angle, did not show differences between the two groups. While a higher Q angle has been implicated as a contributing factor in PFP, our findings did not support this relationship [39]. Although some authors have reported a higher Q angle in women with patellofemoral pain, our study did not confirm this [32,38].

These findings suggest that patellofemoral pathology may not be linked to specific anatomical features within the PFP population. Instead, the biodynamic component may serve as a risk factor for the development of the condition, as dynamic stabilizers, such as the quadriceps, and static stabilizers play a crucial role [7]. Assessing the muscular component is vital in our study, as it contributes significantly to determining the medial/lateral balance [36].

While previous studies have documented weakness in various muscle groups, our research specifically evaluated the effects of aerobic and impact physical activity on muscle strength [4,9,16,32,38,39,40,46]. Analysis of the control group revealed a significant increase in strength for the hip abductors, knee extensors, and performance on the Single-Leg Triple-Hop (SLTH) test after exercise. Conversely, the PFP group also showed increases in strength and distance, except for hip abductors, which continued to exhibit weakness. The observed differences in muscle behavior between the two groups suggest that the muscular component may play a critical role in patellofemoral pain. Previous studies have generally reported decreased strength in hip abductors, extensors, external rotators, and knee extensors [4,9,16,32,38,39,40,46].

When comparing pre-exercise measurements between the two groups, the recorded values were consistently higher in the control group. The only measurement that was greater in the PFP group was the hamstring-to-quadriceps (H/Q) ratio, averaging 0.86 compared to 0.78 in the control group. This ratio in the PFP group is considered abnormal, as the recommended value for the average population is 0.66, with limits approaching 0.5 and 0.80 [47,48]. Kellis et al. [49] noted that a 15% difference in muscle strength between populations increases the risk of injury by 4.66 times. In our study, the PFP group exhibited only a 10% difference, indicating no elevated risk of injury.

The comparison of post-exercise measurements between the control and PFP groups revealed significant differences in hip abductor strength, knee extensor strength, and SLTH test performance, with higher values observed in the control group. A critical variable to discuss in our study is the hip abductors, which, unlike other muscle groups, demonstrated a decrease in strength rather than an increase. Several authors have noted that the femur internally rotates to enhance the power of the hip abductors when necessary [46,50]. The persistent weakness and tendency for further strength reduction during exercise predispose the femur to internal rotation, compensating for dysfunction and attempting to restore abduction force as much as possible. This internal rotation of the femur can lead to lateral patellar displacement and increased joint stress [51].

Although the H/Q ratio did not show a significant difference between the two groups, the PFP group’s ratio indicated an imbalance between the hamstrings and quadriceps. In contrast, the control group’s H/Q ratio showed a slight increase, nearing a tolerable value of 0.79 [48]. This suggests that, despite the presence of an imbalance, it becomes more pronounced and less controlled after aerobic activity, although the differences were not statistically significant. Abnormal H/Q values are associated with lower dynamic knee stability, weaker quadriceps torque, and patellar malalignment [52,53].

The HADD/HABD ratio exhibited non-significant values in both groups before and after aerobic activity, with both groups scoring below 0.56. Ideal values for athletic populations are reported to be close to 0.80, and values below this threshold are associated with a higher risk of injury. There are currently no established ideal values for the general population [45,46].

Several limitations of the study should be considered, including the need for examining muscle behavior under isometric, concentric, and eccentric gym exercises to determine which provides the most significant benefits and balance. The absence of a gold-standard test and the variability of exercise types, durations, and intensities may lead to different results regarding muscle strength and joint behavior pre- and post-exercise, highlighting the need for careful consideration in future studies. The use of ultrasound could serve as a valuable diagnostic tool, especially given the increased circumference measurement above the patella, which may indicate bursitis or joint effusion.

Additionally, the groups could have been better designed with a larger sample size and a focus on a single gender to facilitate more meaningful comparisons. While the study concentrated on evaluating muscle strength, gender was not considered a relevant factor during planning. Another limitation was the lack of interobserver evaluations, which could have improved the measurement of various angles obtained from X-Rays, thereby enhancing result validity. To address this, we had the radiologist measure the images three times on different occasions, yielding an average value.

Ultimately, since a significant percentage of patients continue to experience persistent symptoms despite various management strategies, conducting more evaluative and therapeutic studies is essential for a deeper understanding of the pathophysiology of this condition.

## 5. Conclusions

The evaluation of muscle strength revealed significant alterations in hip abductors and knee extensors when comparing the two groups. Specifically, the hip abductors exhibited a decrease in strength following exercise, whereas the control group demonstrated a tendency for improvement. Additionally, the H/Q ratio in the PFP population was below the desired threshold and tended to worsen after the physical test. A comprehensive assessment of limb mechanics can assist physicians in determining the most appropriate therapeutic approach. However, other variables examined did not show significant correlations with patellofemoral pain.

## Figures and Tables

**Table 1:** Demographic characteristics of the study subjects.

Variable	PFP (*n* = 17)	Control (*n* = 17)	*p*-Value *
Gender (*n*)	Male (4), Female (13)	Male (4), Female (13)	
Age (years)	22.9 ± 3.9	24.9 ± 4.2	0.159
Height (m)	1.65 ± 0.01	1.62 ± 0.07	0.420
Weight (kg)	67.2 ± 12.7	64.5 ± 11.6	0.523
BMI (kg/m^2^)	24.4 ± 2.6	24.2 ± 3.1	0.816

Data are presented as mean ± standard deviation. BMI, body mass index; PFP, patellofemoral pain. * Unpaired *t*-test.

**Table 2:** Anthropometric characteristics of the study subjects.

Variable	Group		*p*-Value *
	PFP	Control	
Iliac superior spine distance (cm)	25.0 ± 1.5	24.9 ± 2.7	0.8752
Thigh circumference at the superior pole of the patella (cm)	39.7 ± 2.8	37.7 ± 2.3	0.0300
Thigh circumference 5 cm superior to the pole of the patella (cm)	42.8 ± 3.2	40.9 ± 3.0	0.0880
Thigh circumference 10 cm superior to the pole of the patella (cm)	47.3 ± 3.9	46.2 ± 2.9	0.3587

Data are presented as mean ± standard deviation. PFP, patellofemoral pain. * Unpaired *t*-test.

**Table 3:** Clinical scale scores of the study subjects.

Questionnaire	Group		*p*-Value *
	PFP	Control	
IKDC	63.6 ± 12.9	99.5 ± 1.6	<0.0001
Kujala	75.2 ± 10.8	99.3 ± 1.7	<0.0001
SF-12 Physical	43.5 ± 9.7	57.1 ± 1.8	0.0003
SF-12 Mental	52.3 ± 13.4	53.9 ± 8.7	0.7042
KOOS	67.5 ± 12.3	98.8 ± 3.6	<0.0001
KOOS Symptoms	72.8 ± 12.6	99.3 ± 1.6	<0.0001
KOOS Pain	67.1 ± 12.4	98.5 ± 6.1	<0.0001
KOOS Daily Living	84.7 ± 10.5	100.0 ± 0.0	<0.0001
KOOS Sports	61.2 ± 24.1	99.1 ± 2.0	<0.0001
KOOS Quality of Life	51.2 ± 18.7	98.9 ± 4.6	<0.0001

Data are presented as mean ± standard deviation. IKDC, International Knee Documentation System; SF-12, Short Form Health Survey; KOOS, Knee Injury and Osteoarthritis Outcome Score; PFP, patellofemoral pain. * Mann–Whitney test.

**Table 4:** Results of the radiological measures in the study subjects.

Radiographic Variable	Group		*p*-Value *
	PFP	Control	
Merchant angle 30° degrees	9.0 ± 5.9	7.1 ± 3.6	0.2650
Sulcus angle 30° degrees	136.8 ± 3.8	132.5 ± 5.6	0.0140
Laurin angle 30° degrees	12.5 ± 3.2	18.9 ± 19.0	0.1817
Merchant angle 60° degrees	6.3 ± 4.3	5.4 ± 2.1	0.4204
Sulcus angle 60° degrees	137.2 ± 5.2	135.4 ± 4.6	0.2897
Laurin angle 60° degrees	15.7 ± 6.1	16.7 ± 4.6	0.5852
Merchant angle 90° degrees	4.9 ± 1.6	5.7 ± 1.7	0.1657
Sulcus angle 90° degrees	140.8 ± 4.5	139.0 ± 4.2	0.2395
Laurin angle 90° degrees	15.7 ± 3.3	17.8 ± 3.6	0.0893
Tibio–calcaneal angle	15.5 ± 4.8	13.2 ± 5.5	0.1978
Mechanic external femoral angle	88.8 ± 3.2	90.4 ± 2.5	0.1044
Anatomic external femoral angle	84.0 ± 4.0	82.9 ± 1.9	0.3130
Medial proximal tibial angle	88.2 ± 2.0	88.6 ± 2.5	0.6058
Q angle	12.8 ± 9.1	11.9 ± 6.4	0.7463

Data are presented as mean ± standard deviation. PFP, patellofemoral pain. * Unpaired *t*-test.

**Table 5:** Muscle strength evaluation and SLTH test scores of the study subjects.

Variable	PFP		Control		*p*-Value *
	Pre	Post	Pre	Post	
Hip Flexor (N⋅m)	28.2 ± 6.6	28.5 ± 6.7	30.3 ± 7.9	32.5 ± 8.6	0.6781
Hip Extensor (N⋅m)	33.0 ± 13.3	46.9 ± 49.8	34.2 ± 8.0	36.5 ± 8.5	0.6124
Hip Abductor (N⋅m)	39.2 ± 5.1	37.9 ± 7.1 ^a^	42.9 ± 4.6	45.6 ± 7.7 ^a^	0.0007
Hip Adductor (N⋅m)	20.5 ± 5.3	21.4 ± 4.7	22.5 ± 3.7	24.2 ± 5.0	0.1229
Knee Flexor (N⋅m)	28.2 ± 6.6	31.2 ± 8.2	34.4 ± 7.1	36.5 ± 8.1	0.1087
Knee Extensor (N⋅m)	33.6 ± 7.7	36.0 ± 9.1 ^a^	44.9 ± 10.3	47.7 ± 14.0 ^a^	0.0001
H/Q Ratio	0.86 ± 0.15	0.89 ± 0.22	0.78 ± 0.13	0.79 ± 0.14	0.1602
HADD/HABD Ratio	0.52 ± 0.09	0.56 ± 0.09	0.53 ± 0.08	0.54 ± 0.12	0.5467
SLTH Test (cm)	328.9 ± 77.6	337.9 ± 74.9 ^a^	392.2 ± 67.1	438.6 ± 65.8 ^a^	<0.0001

Data are presented as mean ± standard deviation. SLTH, Single-leg Triple-Hop; H/Q, Hamstrings/Quadriceps; HADD/HABD, Hip Adductors/Hip Abductors; PFP, patellofemoral pain; ns, non-significant. * One-way analysis of variance, Tukey’s Multiple Comparison Test. ^a^ Significant differences after comparison of values from the Post-PFP vs. Post-Control groups (*p* < 0.05).

## Data Availability

The datasets used and/or analyzed during the current study are available from the corresponding author on reasonable request.
